# Idiopathic sclerosing orbital inflammation mimicking a malignant spindle cell tumor in a dog

**DOI:** 10.1002/ccr3.639

**Published:** 2016-08-23

**Authors:** Nina Marie Rzechorzek, Colin Smith, Tobias Schwarz, Tiziana Liuti, Richard Elders, Samantha Woods, Jessica Lawrence, Katia Marioni‐Henry

**Affiliations:** ^1^ Hospital for Small AnimalsRoyal (Dick) School of Veterinary Studies and Roslin InstituteUniversity of EdinburghRoslinMidlothianUK; ^2^ Centre for Clinical Brain SciencesUniversity of EdinburghEdinburghMidlothianUK

**Keywords:** Case report, exophthalmos, idiopathic orbital inflammation, IgG4, lymphoplasmacytic infiltration, magnetic resonance imaging, radiation therapy, retrobulbar

## Abstract

A dog presented with a retrobulbar mass, diagnosed histopathologically as malignant spindle cell neoplasia. Emergence of analogous findings in the contralateral orbit prompted extended immunohistochemistry of the original mass and reassignment to idiopathic sclerosing orbital inflammation. Early incisional biopsy with extended immunohistochemical analysis should be considered for canine orbital tumors.

## Case History, Examination, and Differential Diagnosis

A 7‐year‐old, entire male crossbreed dog presented with an 11‐month history of diffuse, left episcleritis. This was initially mild and had been managed by the primary veterinarian with 1% prednisolone acetate ophthalmic suspension. Despite a good therapeutic response, the inflammation repeatedly recurred after each course of treatment. Over the course of 7 months, the condition progressed to unilateral exophthalmos alongside clinical signs consistent with pain including blepharospasm and self‐trauma. The dog was referred to a veterinary ophthalmologist at which point intraocular pressures (OS 16 mmHg; OD 18 mmHg), Schirmer Tear Tests, and visual assessments were within normal limits. Oral prednisolone (0.39 mg/kg PO, q12 h) treatment induced transient clinical improvement, but lateral deviation of the left globe ensued over the subsequent 4 months. Periorbital ultrasonography revealed a soft tissue lesion in the left medial retrobulbar space and on suspicion of an abscess or neoplasm, the dog was referred to the University of Edinburgh Hospital for Small Animals.

On presentation, left exophthalmos, lateral strabismus, brown‐tinged epiphora, and diffuse scleral congestion were evident. The globe was soft on palpation but resistant to retropulsion. An acute pain response was not elicited during globe manipulation or upon mouth opening, inconsistent with the typical presentation of a retrobulbar abscess in the dog. Direct and consensual pupillary light reflexes and menace responses were normal OU. Other clinical examination findings were unremarkable. Differential diagnoses included neoplasia, cyst, infection (cellulitis or abscess), noninfectious inflammatory disease, or foreign body draining tract. The insidious onset of signs with absence of fever and painless palpation decreased the likelihood of an infectious etiology. Although globe retraction (a clinical sign of ocular pain in the dog) could not be assessed because of the retrobulbar mass, there was also no behavioral expression of photophobia. Some level of discomfort was however presumed on the basis of inflammatory signs.

## Investigations, Treatment, and Outcome

**Figure 1:**
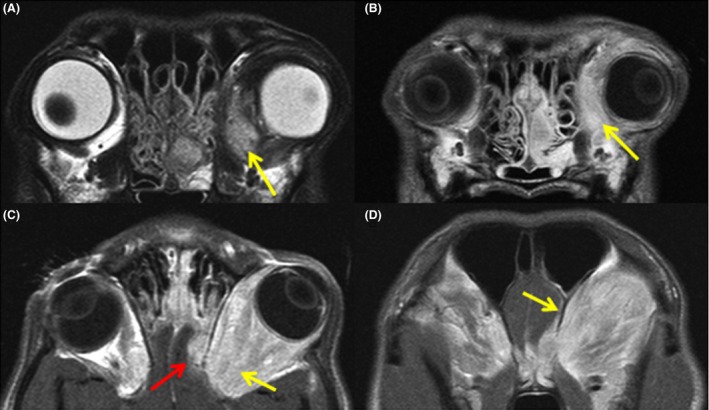
MRI of the head highlighting the mass in the left retrobulbar and periorbital region. (A) Mildly hyperintense signal on transverse T2w sequence (yellow arrow). (B) Moderate to marked contrast‐enhancing signal on transverse T1w postgadolinium sequence (yellow arrow). The medial aspect of the left nasal cavity is partially filled with noncontrast‐enhancing material. (C) Moderate to marked contrast‐enhancing signal on dorsal T1w postgadolinium sequence (yellow arrow). A poorly circumscribed, extra‐axial contrast‐enhancing mass appears to breach through the ethmoid bone, compressing the ventrolateral aspect of the left olfactory bulb (red arrow); this mass appeared to be contiguous with the retrobulbar mass and the nasal hyperintensity. (D) Marked meningeal enhancement surrounding the lateral aspect of the left olfactory bulb (yellow arrow) on a more caudal slice of transverse T1w postgadolinium sequence.

Written informed consent was obtained from the owner for all procedures performed. A complete blood count with biochemistry and coagulation profile revealed only an increase in alkaline phosphatase at 237 U/L (20–60 U/L; anticipated in respect of recent prednisolone therapy) and a mild increase in creatine kinase at 259 U/L (50–200 U/L). Cultures of ultrasound‐guided aspirates of the mass (for aerobic and anaerobic bacteria, mycoplasma, and fungi) produced no growth; May–Grünwald–Giemsa (MGG) cytology of the same aspirates yielded only keratin bars and keratinaceous debris and was considered nondiagnostic. Magnetic resonance imaging (MRI) of the head was performed under general anesthesia at 1.5T, acquiring multi‐sequence images in transverse (T2‐weighted [T2w], T1‐weighted [T1w], T1w postgadolinium, T1w fast field echo) and dorsal (T1w postgadolinium and short tau inversion recovery) planes. Findings (Fig. 1) included marked contrast‐enhancing swelling of the left retrobulbar and periorbital space with lack of fat definition and rostral displacement of the globe. The mass appeared to breach through the ethmoid bone, compressing the ventrolateral aspect of the left olfactory bulb (with focal meningeal contrast enhancement); there was also involvement of the left nasal cavity which was partially filled with noncontrast‐enhancing material. The left zygomatic salivary gland and left mandibular and medial retropharyngeal lymph nodes were enlarged. A mildly hyperintense signal was noted on T2w and postcontrast images of the most caudoventromedial aspect of the left temporalis muscle. Analysis of cerebrospinal fluid (obtained from the cisterna magna) revealed an increased protein concentration (0.79 g/L [<0.45 g/L]) with normal cytology and total cell count, consistent with central nervous system neoplasia, injury, degeneration, or ischemia. Specifically, no etiological agents or exfoliating neoplastic cells were observed, and there was no evidence of meningeal inflammation. Fine needle aspiration and MGG cytology of the left mandibular lymph node indicated reactive lymphoid hyperplasia, neutrophilia, and suspected histiocytosis; aspirates of the right mandibular lymph node were nondiagnostic. Together with the available literature on orbital disorders in canines, the findings were most consistent with a retrobulbar meningioma or soft tissue neoplasm with intranasal and intracranial extension, rhinitis, reactive sialadenitis, and reactive or metastatic local lymphadenopathy and myositis. However, lymphatic drainage from an area of primary chronic inflammation could not be completely excluded.

On the basis of our most likely differential, the dog was referred internally to the Oncology Service 7 days later, by which point there was marked left ocular congestion, corneal ulceration, and constant blepharospasm. Three‐view thoracic radiographs were taken to complete staging and no gross metastatic disease was observed. Left enucleation was elected as a diagnostic and therapeutic measure and required a complex transpalpebral approach with frontal craniectomy. The retrobulbar mass was observed extending deep into the orbit and appeared to be invading the skull in the region of the ethmoidal foramina. The lesion was successfully debulked prior to gentle dissection of the abnormal tissue just lateral to the olfactory bulb – this was removed with part of the cribriform plate and olfactory nerves, followed by lavage of the site and routine closure. Standard intra‐ and peri‐operative analgesia and antibiosis were provided.

**Figure 2:**
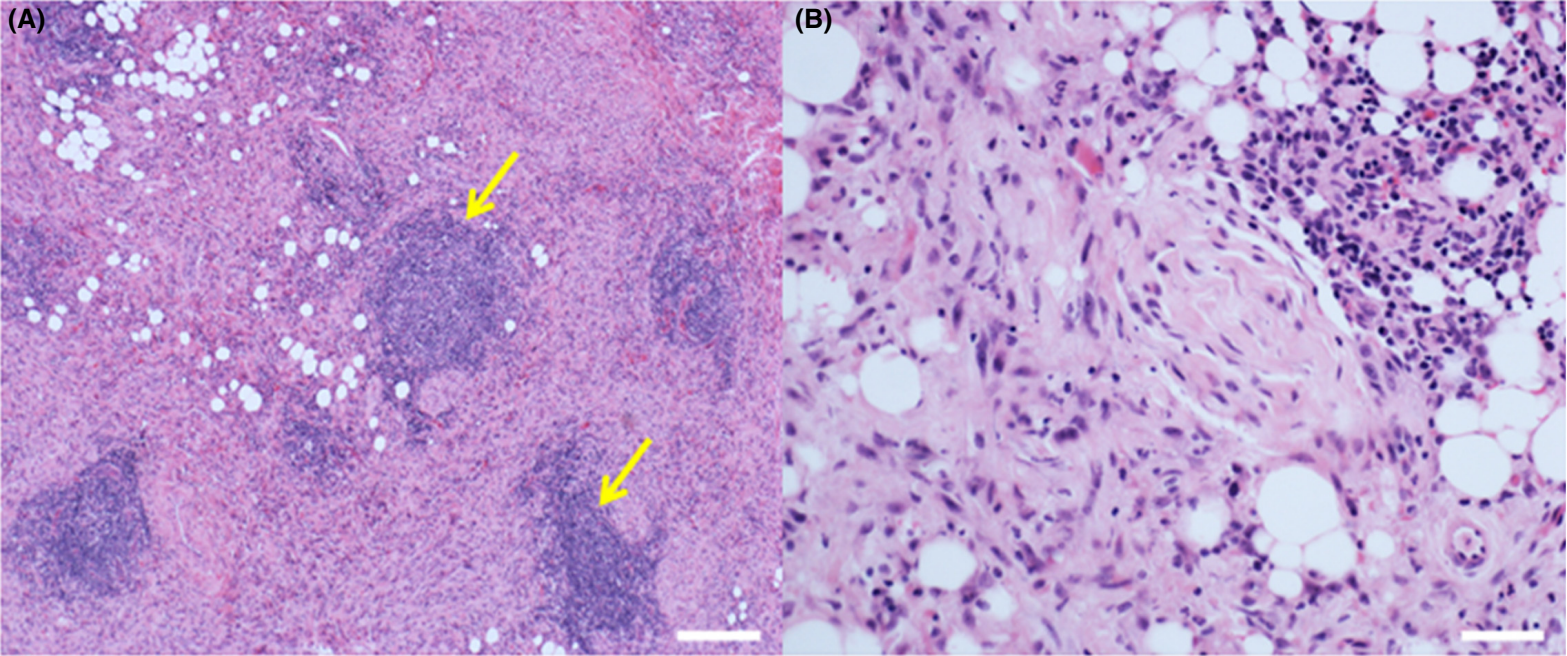
Photomicrographs capturing key histological features of the left retrobulbar mass (hematoxylin and eosin stain). Spindle cell proliferation with lymphoplasmacytic inflammatory nodules (yellow arrows; lacking germinal follicular architecture) and diffuse inflammation infiltrating muscle and adipose tissue. (A) Scale bar = 200 *μ*m (B) scale bar = 50 *μ*m.

Histopathologically, the retrobulbar mass contained skeletal muscle and adipose tissue diffusely infiltrated by a nonencapsulated spindle cell tumor with a fascicular (bundled) architecture lacking storiform structure (a matted, irregularly whorled appearance typical of fibrous histiocytoma) (Fig. 2). The spindle cells showed moderate nuclear pleomorphism, although mitotic figures were relatively sparse (<1 per 10 high‐powered fields at 40X) and there was no intrinsic tumor necrosis. Myofiber loss and regenerative changes were evident. There was a prominent inflammatory infiltrate, consisting of diffuse inflammation interspersed with inflammatory nodules (lymphoplasmacytic aggregates). The tumor also extended into mature cancellous and cortical bone of the skull. The spindle cell proliferation expanded and effaced the retrobulbar soft tissue and skeletal muscle. Together with lymphoplasmacytic infiltration and a prominent reactive fibroblastic profile, the aggressive nature of this mass was consistent with a malignant spindle cell tumor, a sclerosing form of idiopathic orbital inflammation (ISOI) or an inflammatory myofibroblastic tumor (IMT) 1, 2, 3. Given the sparcity of literature describing IMT in the dog, the absence of any reports describing ISOI in this species, and the distribution of the lesion, the most likely histological diagnosis was malignant peripheral nerve sheath tumor.

Due to incomplete excision of the suspected retrobulbar sarcoma, definitive‐intent radiation therapy was recommended to decrease the likelihood of tumor recurrence. Radiation therapy consisted of 19 fractions of 2.8 Gy prescribed to the planning target volume (PTV) to a total dose of 53.2 Gy, normalized to 100% in isocenter. The clinical target volume (CTV) included all residual tumor tissue, postsurgical swelling, and 3–5 mm radially to account for microscopic disease. The PTV was created following an expansion of the CTV by 3 mm in all dimensions. Tissue equivalent material (5 mm) was utilized to ensure adequate dose to the superficial subcutaneous tissues. The minimum dose and heterogeneity within the PTV and CTV were 49.3 Gy and 14% and 50.1 Gy and 12%, respectively; the maximum dose was contained within the PTV. The dog tolerated radiation therapy well, although he developed acute grade 2 skin toxicity with erythema and dry desquamation and grade 3 oral mucositis 4. These signs were effectively managed with a standard combination of antiinflammatories, analgesics, antipruritics, antibiotics, and topical treatments, and resolved within 3 weeks.

**Figure 3:**
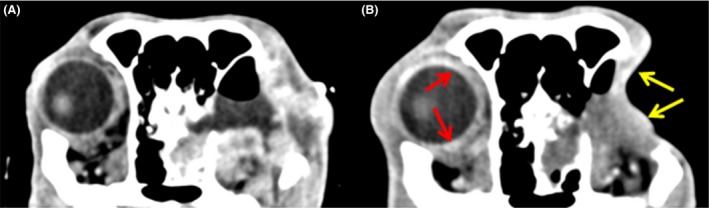
Computed tomography images of the head, postresection of left retrobulbar mass; soft tissue window postcontrast (iohexol). (A) Immediately postsurgery for radiation therapy planning, showing local inflammation around the surgical site. (B) 20 weeks postsurgery. Thickened subcutaneous tissue can be seen within the irradiated field with mild contrast enhancement (yellow arrows) but no gross tumor recurrence. The regional lymph nodes, left eye socket, left orbital bone lysis, seroma, and soft tissue lesion within the left nasal cavity are static compared to (A). There is evidence of reduced local inflammatory reaction (lack of contrast enhancement) in the left retro‐orbital region. Note, however, mild contrast uptake in the right orbital rim – potentially a sign of early inflammation developing on this side (red arrows).

**Figure 4:**
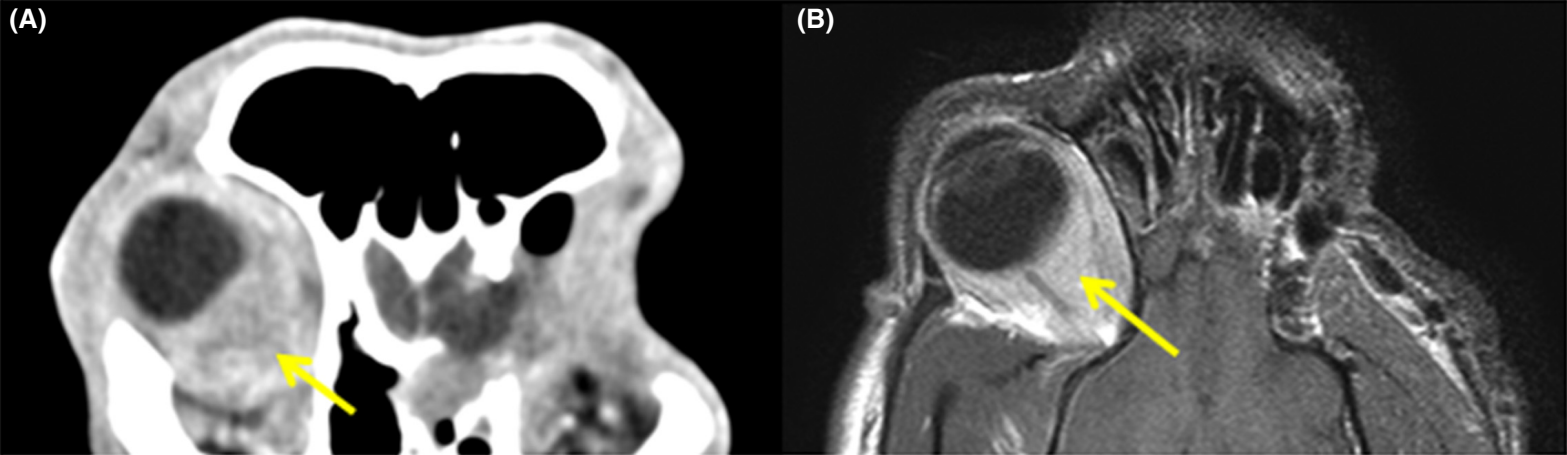
Contralateral emergence of identical retrobulbar lesion 26 weeks postsurgery, identified with advanced imaging. (A) CT (soft tissue window postcontrast) shows right retrobulbar soft tissue mass (yellow arrow), lacking fat intensity, with right temporal and pterygoid myositis. (B) Concordant findings on MRI (dorsal T1w postgadolinium sequence; yellow arrow). The following additional sequences were also performed in transverse (T1w, T2w, T1w postgadolinium, T2w*, fluid‐attenuated inversion recovery), sagittal (T2w), and dorsal (T2w) planes (data not shown).

**Figure 5:**
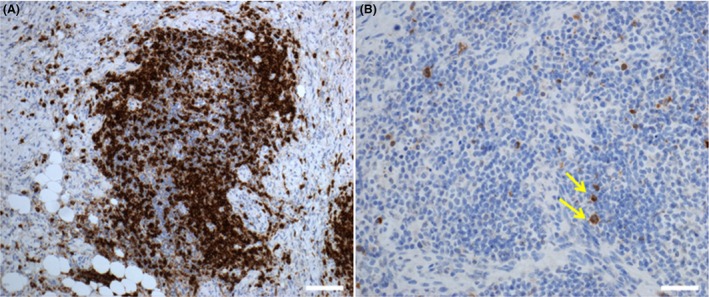
Photomicrographs capturing key immunohistochemical features of the left retrobulbar mass. (A) The inflammatory infiltrate (brown‐stained cells) was predominantly composed of T lymphocytes (CD3 immunolabel; scale bar = 50 *μ*m). (B) Small numbers of IgG4‐expressing plasma cells were noted (brown‐stained cells highlighted by yellow arrows; IgG4 immunolabel; scale bar = 50 *μ*m).

Twenty weeks postsurgery, the dog was clinically well and restaging with computed tomography (CT) provided no evidence of local tumor recurrence (Fig. 3). The regional lymph nodes, left eye socket, left orbital bone lysis, seroma, and soft tissue lesion within the left nasal cavity were static compared to images acquired immediately postsurgery. There was, however, mild contrast uptake in the right orbital rim and 26 weeks postsurgery, the dog represented to the referring veterinarian with right ocular discomfort, right‐sided head tilt, and vacant episodes. Meloxicam (0.1 mg/kg PO, q24 h) and 1% prednisolone acetate ophthalmic suspension (one drop in the affected eye, q6–8 h) failed to resolve all of the signs (although the head tilt and vacancy disappeared, suggesting that these signs were related to pain rather than to lesions of the vestibular system or forebrain, respectively). The dog returned to the hospital with right exophthalmos, conjunctival hyperemia, mild episcleral congestion, and ulcerative keratitis. Pupillary light reflex, menace response, lacrimation, and intraocular pressure (19 mmHg) were normal, digital retropulsion was reduced, and ultrasonography revealed a heterogenous mass ventromedial to the orbital rim. Advanced imaging demonstrated similar findings to those observed in the left retrobulbar space at initial referral. A right retrobulbar soft tissue mass (lacking fat intensity), with right temporal and pterygoid myositis was observed with both MRI and CT, but no evidence of the original tumor was seen (Fig. 4). Immunosuppressive therapy (prednisolone at 2 mg/kg PO, q24 h for 7 days, then switching to dexamethasone at 0.3 mg/kg PO, q24 h) temporarily relieved the clinical signs, but the dog died 9 days later due to clinical signs unrelated to the ocular mass and further investigations including necropsy were declined. Retrospective immunohistochemical labeling (Table 1) of the left retrobulbar mass was negative for S100, CD34, bcl2, desmin, MyoD, and smooth muscle actin, ruling out IMT 2, 5 and most spindle cell sarcomas including a peripheral nerve sheath tumor. The inflammatory infiltrate was composed predominantly of T lymphocytes (CD3), with small numbers of plasma cells (CD138), a subset of which expressed IgG4 (Fig. 5). However, the lesion did not fulfill suggested histological criteria for IgG4‐related disease (including an IgG4+/IgG+ plasma cell ratio of >40%) and the final diagnosis was most consistent with ISOI 6, 7.

**Table 1:** Details of immunohistochemical methods

Antibody	mAb^a^/pAb^b^	Dilution	Blocking	Pretreatment	Supplier
S100	pAb	1:1500	Peroxide	Enzyme	Dako
CD34	mAb	1:50	Peroxide	EDTA^c^	Dako
Bcl2	mAb	1:50	Peroxide	EDTA	Dako
Desmin	mAb	1:100	Peroxide	EDTA	Dako
MyoD	mAb	1:50	Peroxide	Citrate	Dako
SMA^d^	mAb	1:1000	Peroxide	EDTA	Dako
CD3	mAb	1:200	Peroxide	EDTA	Leica
CD138	mAb	1:100	Peroxide	EDTA	Dako
IgG4	mAb	1:8000	Peroxide	EDTA	TBS^e^

a Monoclonal antibody.

b Polyclonal antibody.

c Ethylenediaminetetraacetic acid.

d Smooth muscle actin.

e The Binding Site.

## Discussion

We have reported the progressive clinical course and key diagnostic features of a novel, noninfectious inflammatory orbital disease in a dog. Such disorders occur infrequently in animals and include immune‐mediated conditions and idiopathic orbital inflammation (IOI; otherwise known as orbital pseudotumor) 1, 2, 8. We consider that the term “pseudotumor” is less than ideal for these orbital inflammatory growths – we have thus adopted the most recent nomenclature from the human literature to promote cohesive cross‐translational understanding of the pathology while awaiting a more refined classification in companion species. Most orbital tumors are considered to be malignant with a poor to guarded prognosis 9, 10; by contrast, IOI is defined as benign, orbital inflammation without evidence of a specific local or systemic etiology 8, 11, 12, 13, 14. A common disorder in humans, IOI occurs most often between the third and fifth decades of life 8, 11, 13, 15. These lesions may be bilateral or alternating (ipsilateral remission followed by contralateral emergence) but typically present unilaterally, thus mimicking neoplasms 8, 15, 16, 17. Although nonmetastatic, IOI is locally aggressive, often leading to severe visual and oculomotor dysfunction, requiring surgical intervention 12, 13. Having excluded a specific cause for the dog's presenting signs, and noting the late mimicry of these signs in the right orbit, we considered IOI as a differential diagnosis.

Other differentials for progressive inflammatory lesions leading to exophthalmos in the dog include nodular granulomatous episcleritis (NGE), nodular fasciitis, and fibrous histiocytoma 9. Nodular granulomatous episcleritis typically affects the cornea or adnexal structures such as the temporal limbus and nictitating membrane and is often bilateral, although the single published report of orbital NGE in the dog had a unilateral presentation 9. Likewise, canine ophthalmic nodular fasciitis appears to affect the conjunctiva and eyelids rather than the orbit 18 and we are not aware of any bilateral presentations of this disorder in humans or animals. Unlike the lesion described above, the orbital NGE lesion reported by Barnes et al. 9 appeared to communicate with the nictitating membrane without any bony involvement or extension beyond the orbit. In addition, regional lymphadenopathy was not detected 9. While the retrobulbar mass in this report had a nodular component histologically, it lacked the true lymphoid follicular architecture noted in NGE and was predominated by T lymphocytes rather than B lymphocytes 9. The lack of a moderate eosinophilic component and the aggressive fibrotic proliferation further distinguish this lesion from NGE, and fibrous histiocytoma is ruled out by the absence of histiocytic cells. Nodular fasciitis (also described as pseudosarcomatous fasciitis and nodular fibrositis) rarely affects the orbit and tends to have a more rapid clinical course 19, 20, 21, 22. Like ISOI, its histological features mimic sarcoma and involvement of adipose tissue and skeletal muscle may be observed along with nerve entrapment and some mitotic figures 20. However, most reported cases of nodular fasciitis affecting the eye and adnexa in humans have demonstrated a variable amount of intercellular myxoid ground substance, multinucleated giant cells, and only scant infiltration of lymphocyte and mononuclear cells rather than lymphocytic aggregates 20, 22 – histopathological features distinct from ISOI. In addition, although bony erosion has been reported in a few cases 23, 24, the prognosis for nodular fasciitis is generally excellent after local excision 20, 21, 22.

The left retrobulbar mass described herein penetrated the bony walls of the orbit, and compressed the left olfactory bulb. Likewise, bony destruction occasionally results from severe chronic IOI in humans, which classically involve orbital fibrosis, optic nerve compression, and impaired ocular rotation 15. As noted in the dog, subsequent intracranial infiltration produces local meningeal contrast enhancement on MRI 25. However, in contrast to humans, the canine orbital margin is incomplete, with contributions from the zygomatic salivary gland and medial surface of the temporalis muscle 13. Changes in these structures on MRI may thus represent a manifestation of IOI peculiar to the dog. Although many human IOI lesions appear hypointense on T2w images, the hyperintensity of the canine retrobulbar mass is typical of sclerosing subtypes of human IOI (ISOI) in which active inflammation and edema can mask the effects noted in other types 11, 16. In essence, the canine imaging data effectively mirror that described for human ISOI, excepting minor differences explained by species‐specific anatomy.

Histologically, the canine mass was dominated by patchy aggregates of lymphocytes and fibrous proliferation – akin to late‐stage human IOI lesions in which increased connective tissue is consistently present 15. Descriptions of orbital pseudotumor in the veterinary literature, however, include only one prosimian primate and a lacrimal pseudotumor in a bull terrier 26, 27. Feline lesions originally recorded as orbital pseudotumor have since been reclassified as feline restrictive orbital myofibroblastic sarcoma (FROMS) owing to their highly aggressive nature, resulting in bilateral exenteration and/or euthanasia in all reported cases 1, 3, 28, 29. However, a few early reports in this species did describe a relatively extended survival time alongside immunosuppressive treatment 13, suggesting one or more of the following: (1) that a less aggressive subtype exists, (2) that patient‐derived factors modify disease progression and/or therapeutic response, or (3) that FROMS and feline IOI are two separate disease entities. The course of FROMS resembles the clinical history of the dog, with insidious exophthalmos and progressive lack of globe mobility 1, 3. Neither FROMS nor IOI are reported to metastasize – however, they exhibit divergent microscopic features. Feline restrictive orbital myofibroblastic sarcoma displays a mixed inflammatory response with perivascular cellular infiltrate and fibrous tissue spreading along fascial planes 1. Mild fibroblastic pleomorphism without mitotic figures and positive immunohistochemistry for S100, smooth muscle actin and vimentin (with or without glial fibrillary acidic protein) are additional characteristic features not seen in the canine mass 1, 28.

Idiopathic sclerosing orbital inflammation accounts for 6–8% of human inflammatory orbital lesions and is otherwise described as “sclerosing orbital pseudotumor” 11, 16, 30. ISOI patients exhibit fewer inflammatory signs, a more chronic onset, and more aggressive disease course than other IOI types, reminiscent of that seen in the dog 30. Histopathologically, ISOI comprises a dense fibrosis with a paucicellular infiltrate of lymphocytes, histiocytes, eosinophils, and plasma cells 30, 31. This damages orbital structures via cicatricial entrapment with mass effect and is associated with a poor response to conventional therapy (corticosteroids ± radiation) 11, 17, 25, 30, 31. While several of these features may be seen with aggressive orbital neoplasms, we are not aware of any neoplastic disorders that would present independently (and almost identically) in both orbits. Overall therefore, the clinical, radiological, and immunohistochemical features of the lesion in this dog are most consistent with ISOI.

A small proportion of IgG4‐positive plasma cells were identified within the ISOI which, to the best of our knowledge, is the first reported observation of this cell subclass within a pathological lesion affecting the canine orbit. Recently characterized, human IgG4‐related disease comprises a group of fibro‐inflammatory conditions of unknown origin featuring tumor‐like expansions in one or multiple organs, with sclerosing fibrosis and a dense lymphoplasmacytic infiltrate rich in IgG4‐positive plasma cells 6, 7. A significant proportion of human ISOI cases are now known to be IgG4‐related, and importantly, this trait imparts favorable susceptibility to steroid and radiation therapy 31. Although the canine lesion exhibited many characteristics of IgG4‐related ISOI, the relative density of IgG4‐positive plasma cells was insufficient to make this diagnosis based on the proposed human criteria 6, 7. This is consistent with the steroid resistance observed initially, however, we cannot exclude the possibility that early treatment with antiinflammatory doses of prednisolone may have altered the IgG4‐positive component of our immunohistochemical readout in favor of an ISOI diagnosis 31. Many human patients experience recurrence following acute‐phase resolution of IOI with immunosuppressive corticosteroid treatment 3, 17. Unfortunately, in this dog, the long‐term efficacy of the immunosuppressive treatment with corticosteroids could not be evaluated due to his unexpected death. The evidence base is equally unclear for the use of nonsteroidal antiinflammatories, chemotherapeutic agents, and other immunomodulatory drugs in the context of ISOI 2, 12, 17. Although the relative long‐term benefit of radiation therapy cannot be determined in this case, local control of residual tumor tissue in the left orbit was retained for at least 26 weeks.

**Figure 6:**
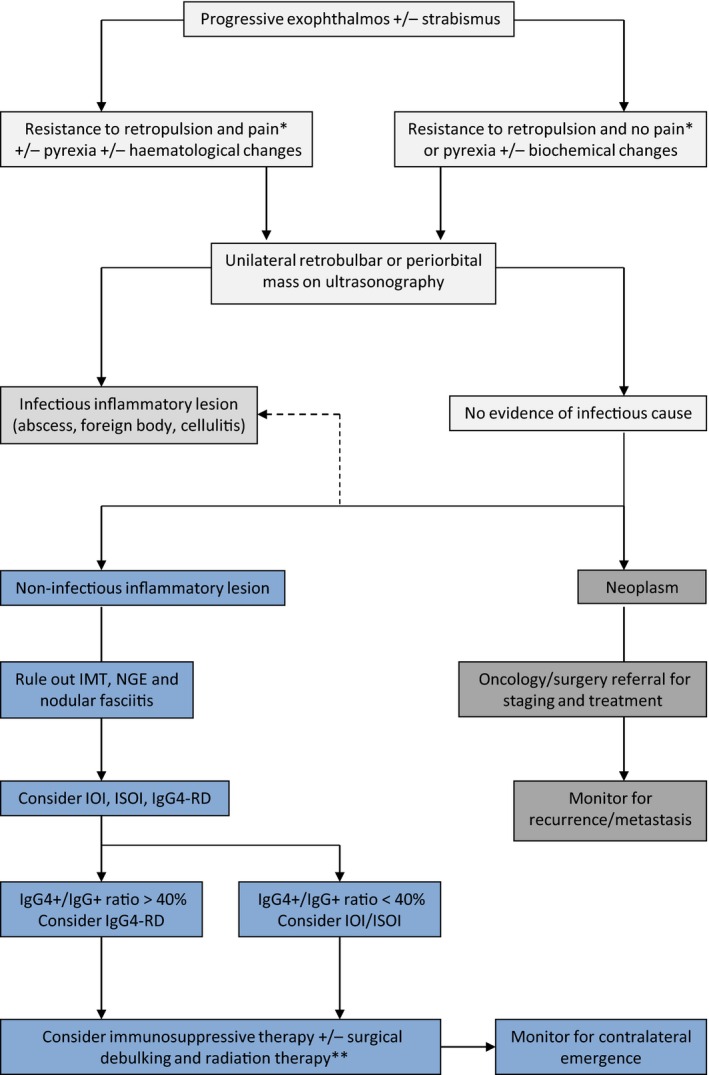
Proposed diagnostic approach to progressive exophthalmos in the dog. Early referral to ophthalmology (and/or neurology) is indicated for progressive exophthalmos. Ultrasonography should include both orbits; upon observation of a retrobulbar mass, ultrasound‐guided FNA for cytology and culture and sensitivity (aerobic/anaerobic bacteria, mycoplasma, and fungi) should be performed. In some geographical locations, a latex cryptococcal antigen agglutination test might be warranted. For infectious inflammatory lesions, blood culture and targeted antimicrobial therapy with or without surgical exploration should be considered. Lack of evidence for an infectious cause should prompt advanced imaging including MRI with contrast and CSF analysis (if MRI indicates intracranial and/or meningeal involvement). At this stage, an incisional biopsy of any soft tissue mass should be performed followed by histopathology, extensive immunohistochemistry, and culture. Incisional biopsy was not considered prior to enucleation and debulking in this case because of (a) the rapid progression of clinical signs and pain that were compromising welfare (b) the consideration that biopsy would also require general anesthesia and (based on our top differential) we anticipated that debulking of the mass would still be required in due course, and (c) that incisional biopsy would have provided less information than excision with respect to diagnosis and staging. In the case of a positive culture, the diagnosis should be revised (dotted arrow). For confirmed neoplasms, three‐view thoracic radiographs and FNA of regional lymph nodes are required for staging. Note that a bilateral presentation should rouse suspicion of a noninfectious inflammatory pathogenesis. For these lesions, the subtype should be determined and IgG4/IgG ratio should be investigated by both serology and immunohistochemical analysis. Monitoring for pain and ulcerative keratitis is paramount throughout the diagnostic work‐up and should be treated promptly. *Signs of ocular pain in the dog include photophobia, blepharospasm, self‐trauma, increased lacrimation, acute aversive response on palpation of the globe or upon mouth opening and globe retraction (note that globe retraction may be prohibited by a retrobulbar mass). **Enucleation may be required at later disease stages on welfare grounds. IMT, inflammatory myofibroblastic tumor; NGE, nodular granulomatous episcleritis; IgG4‐RD, IgG4‐related disease; IOI, idiopathic orbital inflammation; ISOI, idiopathic sclerosing orbital inflammation.

In conclusion, this report represents the first description of ISOI in an animal, with pathological features distinct from FROMS, IMT, NGE, and nodular fasciitis. Whilst IOI is rare beyond human medicine, our findings highlight the need to consider this diagnosis in dogs presenting with recalcitrant ophthalmic signs associated with a retrobulbar mass. Given their proximity to the brain, and propensity for local invasion and contralateral emergence, early referral for these lesions is warranted, even though a standard‐of‐care therapy is not clear 1, 28, 29. Advanced imaging instituted promptly may capture early changes in the contralateral orbit (for bilateral presentations) before clinical signs emerge, and immunomodulatory therapy (with or without surgery) may improve patient welfare. Most importantly, however, the diagnostic challenge of this case argues for incisional biopsy of orbital masses at first presentation, followed by extended immunohistochemical analysis to rule out chronic inflammation and other benign lesions that mimic neoplastic disease (Fig. 6). Specific stains for inflammatory cell phenotypes and polyclonality of lymphoplasmacytic populations can delineate IOI from lymphoproliferative disease and other neoplasms. Whether ISOI represents a malignant transformation of IOI is worthy of further investigation. On the basis of this case, we propose that ISOI be considered as a differential diagnosis for progressive exophthalmos in the dog.

## Conflict of Interest

None declared.
